# Neuroanatomical Classification in a Population-Based Sample of Psychotic Major Depression and Bipolar I Disorder with 1 Year of Diagnostic Stability

**DOI:** 10.1155/2014/706157

**Published:** 2014-01-19

**Authors:** Mauricio H. Serpa, Yangming Ou, Maristela S. Schaufelberger, Jimit Doshi, Luiz K. Ferreira, Rodrigo Machado-Vieira, Paulo R. Menezes, Marcia Scazufca, Christos Davatzikos, Geraldo F. Busatto, Marcus V. Zanetti

**Affiliations:** ^1^Laboratory of Psychiatric Neuroimaging (LIM-21), Department and Institute of Psychiatry, Faculty of Medicine, University of São Paulo, Dr. Ovídio Pires de Campos Street, 3rd Floor, LIM-21, Nuclear Medicine Center, 05403-010 São Paulo, SP, Brazil; ^2^Center for Interdisciplinary Research on Applied Neurosciences (NAPNA), Nuclear Medicine Center, University of São Paulo, Dr. Ovídio Pires de Campos Street, 3rd Floor, LIM-21, Nuclear Medicine Center, 05403-010 São Paulo, SP, Brazil; ^3^Section of Biomedical Image Analysis (SBIA), Department of Radiology, University of Pennsylvania, 3600 Market Street, Suite 380, Philadelphia, PA 19104, USA; ^4^Laboratory of Neuroscience (LIM-27), Department and Institute of Psychiatry, Faculty of Medicine, University of São Paulo, Dr. Ovídio Pires de Campos Street, 3rd Floor, LIM-27, Institute of Psychiatry, 05403-010 São Paulo, SP, Brazil; ^5^Department of Preventive Medicine, Faculty of Medicine, University of São Paulo, 455 Dr. Arnaldo Avenue, 01246-903 São Paulo, SP, Brazil; ^6^Laboratory of Psychopharmacology and Clinical Psychophysiology (LIM-23), Department and Institute of Psychiatry, Faculty of Medicine, University of São Paulo, Dr. Ovídio Pires de Campos Street, Ground Floor, LIM-23, Institute of Psychiatry, 05403-010 São Paulo, SP, Brazil

## Abstract

The presence of psychotic features in the course of a depressive disorder is known to increase the risk for bipolarity, but the early identification of such cases remains challenging in clinical practice. In the present study, we evaluated the diagnostic performance of a neuroanatomical pattern classification method in the discrimination between psychotic major depressive disorder (MDD), bipolar I disorder (BD-I), and healthy controls (HC) using a homogenous sample of patients at an early course of their illness. Twenty-three cases of first-episode psychotic mania (BD-I) and 19 individuals with a first episode of psychotic MDD whose diagnosis remained stable during 1 year of followup underwent 1.5 T MRI at baseline. A previously validated multivariate classifier based on support vector machine (SVM) was employed and measures of diagnostic performance were obtained for the discrimination between each diagnostic group and subsamples of age- and gender-matched controls recruited in the same neighborhood of the patients. Based on T1-weighted images only, the SVM-classifier afforded poor discrimination in all 3 pairwise comparisons: BD-I versus HC; MDD versus HC; and BD-I versus MDD. Thus, at the population level and using structural MRI only, we failed to achieve good discrimination between BD-I, psychotic MDD, and HC in this proof of concept study.

## 1. Introduction

Mood disorders share a large number of clinical and neurobiological features. The nonspecificity and variability of symptoms over time are frequent causes of misdiagnosis in patients with bipolar disorder (BD) [1, 2]. Although more frequent in BD, psychotic symptoms may be present in some patients with major depressive disorder (MDD) [3]. Nevertheless, epidemiological studies have shown that patients presenting depressive episodes with psychotic features are at increased risk for developing BD [2, 4]. Thus, a medical tool that reliably differentiates patients with psychotic MDD from BD at an early stage of the illness would be highly useful to aid psychiatrists to improve diagnostic accuracy and, consequently, treatment response and prognosis in the clinical practice.

Neuroanatomical pattern classification is a relative new technique that holds promise in solving diagnosis and outcome issues in psychiatry [5]. This new method for brain image analysis allows voxelwise between-group comparisons and classification of scans at an individual basis [5, 6]. Given the multivariate nature of their statistical approach, and the possibility to employ both linear and nonlinear analysis models, these techniques afford improved sensitivity to uncover complex morphological brain differences in comparison to other voxelwise methods [7]. Moreover, once the pattern of abnormalities which better discriminates two groups is defined, this morphological signature can be used to classify images at an individual basis, and measures of diagnostic accuracy (DA) can be obtained [5, 6].

Up until now, a limited number of magnetic resonance imaging (MRI) studies have investigated the usefulness of pattern classification methods in the evaluation of affective disorders, producing variable results. Most studies implemented functional MRI (fMRI) to investigate neuroanatomical classifiers in groups of depressed patients and healthy controls (HC). Such studies have shown diagnostic accuracies (DA) as higher as 82% [8–12]. Regarding BD, investigations are still scarce. In a fMRI study, Costafreda et al. [13] applied a classifier based on support vector machine (SVM) in the discrimination between BD versus schizophrenia (SZ) versus HC and found that SZ were more correctly identified (AD = 92%) than BD (AD = 79%). Three fMRI studies investigated the use of pattern classification approaches to discriminate depressive BD and MDD [14–16]. Although two of those studies [15, 16] have shown good DA (up to 90% in Grotegerd et al. [15]), Mourão-Miranda et al. [14] have found no statistically significant DA to discriminate MDD from depressive BD.

Few studies have investigated the usefulness of pattern classification methods based on structural MRI in mood disorders, with inconsistent results. Liu et al. [17] found that MDD patients with and without resistance to pharmacological treatment could be discriminated from each other, as well as from HC, with fair accuracies as higher as 82%. In a sample of drug-naïve patients with MDD submitted to MRI scans before starting antidepressant treatment, Gong et al. [18] found that grey matter (GM) could discriminate patients from HC with suboptimal DA of 67% (refractory MDD) and 76% (nonrefractory MDD); white matter (WM) was statistically significant only for discriminating nonrefractory patients, with DA of up to 84%. Investigating whole-brain structural neuroanatomy as a diagnostic biomarker, Costafreda et al. [19] obtained a modest DA of 67.6% in the discrimination between MDD patients and HC using a SVM-based classifier. Qiu et al. [20] studied a group of drug-näive patients presenting a first-episode of MDD (*n* = 32) versus HC (*n* = 32) with a SVM classifier and different combinations of morphometric features. The authors reported overall modest classification accuracies ranging from 50% to 78% depending on the combination of features employed [20]. Only one morphometric MRI study has applied pattern classification techniques in BD, comparing two independent samples of patients with BD type I (BD-I) versus HC [21]. The authors found modest DA of up to 73% in the differentiation between BD-I patients versus HC when the classification was performed with the GM and 78% for the analysis based on the WM. However, the two samples were composed of chronic medicated patients, and such results should be interpreted with caution.

Differences in the pipelines for image processing, feature extraction/dimensionality reduction, and pattern recognition methods might at least partly account for the discrepancies observed across studies using pattern classification techniques in neuropsychiatric disorders [6]. Another potential factor associated to this variability of results is the widespread adoption of unsystematic single-diagnosis approach for the definition of the groups under study, which limits the validity of the categories that will be informed to the classifier [22]. An additional issue that might also contribute for the heterogeneity of findings is the occurrence of selection bias. In this regard, it is relevant to note that none of the investigations of affective disorders employing neuroanatomical pattern classification to date have employed population-based approaches. In population-based studies, epidemiological methods are used to identify and recruit representative samples of cases and demographically matched controls from the same, circumscribed geographical area. The use of such designs reduces selection biases by ensuring that control individuals truly represent the population from which the cases came from [23, 24].

To the best of our knowledge, no study to date has investigated the diagnostic performance of a neuroanatomical classifier in the discrimination of patients with a first-episode of mania from individuals presenting their first-episode of psychotic MDD. Moreover, most studies evaluating pattern classification methods in mood disorders to date have used fMRI, which has a of relatively complex implementation and is less available in the clinical practice when compared to 1.5 T structural MRI.

In this proof of concept morphometric MRI study, a sample of individuals with first-episode of psychotic mania (BD-I) and psychotic MDD and a group of demographically matched controls were recruited from the same defined geographical area using an epidemiologic approach. All subjects were followed up naturalistically over a 1-year period, with reinterviews carried out for diagnostic confirmation. A support-vector machine (SVM) classifier was employed to ascertain how distinguishable are BD-I with psychotic features and psychotic MDD at the time of first-presentation using the widely available T1-weighted MRI data. The SVM method applied here has been used in a number of previous investigations of neuropsychiatric disorders, showing consistent results [25–27].

## 2. Materials and Methods

### 2.1. Participants and Design

Patients fulfilling Diagnostic and Statistical Manual for Mental Disorders, 4th edition, (DSM-IV) [28] criteria for a first-episode of mania (BD-I) or a first-episode of psychotic unipolar depression (psychotic MDD) were selected from a large sample of first-episode psychosis individuals who took part in a population-based case-control study investigating the incidence of psychotic disorders in a circumscribed region of São Paulo city, as previously described [29, 30]. In the original epidemiological investigation, cases were identified by active surveillance of all people that made contact for the first time with the mental healthcare services for that region between 2002 and 2005 due to a DSM-IV defined psychotic disorder, regardless of its severity (both outpatients and inpatients were recruited), duration of illness, or compliance to treatment. Patients with psychotic disorders due to a general medical condition or substance-induced psychosis were excluded. The research team provided general guidance to patients but they were referenced to treatment at the health services located in the geographical region where they lived. Both patients and controls were reinterviewed after 1 year of followup for clinical assessment and diagnostic confirmation.

Other inclusion criteria for both cases and controls were (a) current age between 18 and 50 years; (b) residence for 6 months or more in defined geographic areas of São Paulo. The exclusion criteria consisted of (a) history of head injury with loss of consciousness; (b) presence of neurological disorders or any organic disorders that could affect the central nervous system; (c) moderate or severe mental retardation; and (d) contraindications for MRI scanning.

In the present investigation, we included the cases diagnosed as having a first-episode of psychotic mania or a first-episode of psychotic MDD according to the Structured Clinical Interview for DSM-IV (SCID) [31] at the time of initial evaluation and who have shown diagnostic stability (i.e., BD-I and MDD diagnoses) over the 1 year of followup. At baseline, 24 cases initially fulfilled criteria for BD-I with psychotic features (23 for first-episode of mania and 1 for psychotic bipolar depression), and 25 for first-episode of psychotic MDD. Over the follow-up period, from the 25 cases of psychotic MDD initially identified, 3 patients were reclassified as BD-I after presenting manic episodes, 2 as schizoaffective disorder, and 1 as delusional disorder. Thus, the final sample of affective disorders after the 1-year diagnostic reevaluation was formed by the following groups: 27 cases of BD-I (of whom 23 entered the study due to a first-episode of mania) and 19 individuals with psychotic MDD whose diagnosis remained stable over the 1-year follow-up period after the first-episode. Details about the other psychosis cases not included in the present investigation can be found elsewhere [30, 32].

In order to obtain a population-based sample of controls, next-door neighbors matched for age (within five years) and gender with the patients were initially screened to exclude the presence of psychotic symptoms using the Psychosis Screening Questionnaire [33] and interviewed with the SCID for the assessment of other psychiatric disorders. This approach resulted in an initial pool of 94 psychosis-free epidemiological controls eligible for the neuroimaging investigation [32].

Aiming at selecting homogeneous control samples to be used by the classifier against the patients, subsamples of HC (free of any Axis I disorder other than specific phobia, including lifetime substance misuse) matched for gender, age and handedness with psychotic BD-I and MDD subgroups were drawn from the total pool of controls. The matching was performed individually when possible, respecting the following hierarchical rank: gender, age, (within a 2-year range), and handedness. Moreover, as it has been shown that the larger the control sample, the higher the statistical power to detect between-group morphometric abnormalities in MRI studies [23, 34], we tried to select as many controls as possible for each comparison. Therefore, the following pairwise comparisons were carried out:

- first-episode psychotic mania (BD-I) (*n* = 23) versus matched HC (*n* = 33);
- first-episode psychotic MDD (*n* = 19) versus matched HC (*n* = 38).
- first-episode psychotic mania (BD-I) (*n* = 23) versus psychotic MDD (*n* = 19).

Local ethics committees approved the study, and all subjects provided informed written consent.

### 2.2. Clinical Assessment Scales

Both patients and controls were screened for substance use with the Alcohol Use Disorders Identification Test (AUDIT) [35] and the South Westminster Questionnaire [36]; when appropriate, diagnoses of substance use disorders was made using the SCID. A general medical history, including medication use, was obtained directly with each participant or with his/her relatives and also through reviewing of medical records.

All clinical assessment tools, including the SCID, were administered to the participants both at baseline and at the 1-year follow-up evaluation.

### 2.3. Neuroimaging Data Acquisition and Analysis

Imaging data were acquired using two identical MRI scanners (1.5 T GE Signa scanner, General Electric, Milwaukee, WI, USA). Exactly the same acquisition protocols were used (a T1-SPGR sequence providing 124 contiguous slices, voxel size = 0.86 × 0.86 × 1.5 mm, TE = 5.2 ms, TR = 21.7 ms, flip angle = 20, FOV = 22 cm, matrix = 256 × 192 pixels). For the three pairwise comparisons conducted here, the number of subjects (%) acquired using Scanner number 1 are 13 (56.5%) BD-I versus 24 (72.7%) matched HC (*χ* ^2^ = 1.59, df = 1, *P* = 0.208); 10 (52.6%) psychotic MDD versus 25 (65.8%) matched HC (*χ* ^2^ = 0.92, df = 1, *P* = 0.336); and 13 (56.5%) BD-I versus 10 (52.6%) psychotic MDD (*χ* ^2^ = 0.064, df = 1, *P* = 0.801).

All images were visually inspected by an experienced radiologist with the purpose of identifying artifacts during image acquisition and the presence of silent gross brain lesions. Five participants have been excluded from the original neuroimaging investigation on first-episode psychosis from which our sample was drawn due to motion artifacts [30].

**Figure 1:**
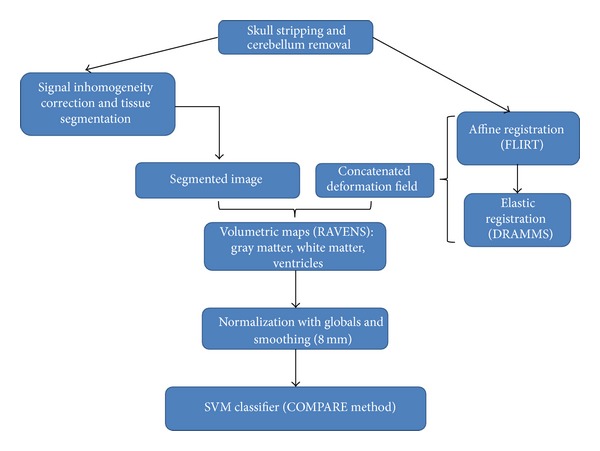
Routine employed for the processing and analysis of T1-weighted MRI images.

The processing and analysis of the structural MRI dataset was performed using a routine previously described by our group [6].  Figure 1 summarizes the pipeline of image processing and analysis employed here.

Initially, the T1-weighted images were preprocessed as follows: skull-stripping; manual removal of the cerebellum in order to improve the tissue segmentation of the temporal lobe; and correction for signal inhomogeneities. The images were subsequently segmented into their 3 principal brain tissue compartments (GM, WM, and cerebrospinal fluid space) through an automated routine. Images were then spatially registered to a Montreal Neurological Institute (MNI) single-subject brain template through two steps (Figure 1). Firstly, an affine transformation was performed using the FLIRT (FMRIB's Linear Image Registration Tool) tool of the FSL (FMRIB Software Library, http://www.fmrib.ox.ac.uk/fsl/flirt) in order to align the major brain structures to the MNI template and also to correct for differences in head positioning. Secondly, a robust method for elastic registration called Deformable Registration via Attribute Matching and Mutual-Saliency weighting (DRAMMS) [37] was employed. The deformation field resulting from the spatial registration of each T1-weighted image to the MNI template was applied to the segmented images in order to generate mass-preserved volumetric maps, named Regional Analysis of Volumes Examined in Normalized Space (RAVENS) maps of the GM, WM, and cerebrospinal fluid compartments [38]. An automated algorithm was used to isolate the cerebral ventricles (lateral ventricles and third ventricle) from the remaining cerebrospinal fluid space, resulting in a ventricular RAVENS map. In the RAVENS maps, the tissue density reflects the amount of tissue present in each subject's image at a given location, after mapping to the standardized template space [38]. Thus, a region of decreased density indicates a reduced volume in this structure, for example. Lastly, the RAVENS maps (GM, WM, and ventricles) were corrected for the total brain volume (given by the sum of all voxels of brain tissue and cerebrospinal fluid space) and smoothed with 8 mm Gaussian kernels.

The GM, WM, and ventricular RAVENS maps were used as inputs for a previously described and validated SVM-based pattern classifier named Classification of Morphological Patterns Using Adaptive Regional Elements (COMPARE) [7] (https://www.rad.upenn.edu/sbia/software/index.html#compare). In this method, voxelwise correlations between RAVENS maps and group membership are used to identify voxels that are candidates to be useful for intergroup discrimination. To achieve the necessary dimensionality reduction, a watershed segmentation algorithm is then used to group voxels into regional clusters and to identify the most relevant features to classification (group discrimination) [7]. This approach also works as an initial feature selection step, reducing the initial dimensionality of the data from millions of variables to a relatively small set of regional volumetric measurements, which the subsequent classifier can handle successfully. In order to improve the spatial consistency of the watershed-derived regional volumetric elements and also to minimize the inclusion of voxels not relevant for the classification (which might reduce the discriminative power), the degree of agreement among all features in its spatial neighborhood is computed by an intraclass correlation coefficient, and a region-growing method based on the Pearson correlation coefficient is employed [7]. Here, the voxel with the highest discriminative power in each watershed-derived region is first selected, and the neighboring voxels are included as long as their inclusion will not decrease the discriminative power of the regional feature. Finally, a feature-selection technique based on SVM criteria is used to select a subset of the top-ranked features that optimizes the performance of the classifier, constituting the “morphological signature” of each group under study which is used by the classifier [7]. The COMPARE classifier, then, employs a nonlinear SVM method to assign a class label to each image under study (individual classification of the MRI scans) through a Gaussian radial basis function kernel.

Although other theoretical frameworks for pattern recognition analyses are available [10, 21], SVM with sufficient dimensionality reduction is currently one of the most widely employed pattern classification models in the study of neuropsychiatric disorders [5, 6]. SVM is a powerful pattern classification method that works to find a line or “decision boundary” that better separates two groups [39]. This boundary may be depicted either by a hyperplane—in the case of linear classifiers—or by a more general hypersurface—when a nonlinear SVM is used—in the high-dimensional feature space where the vectors representing each brain under study are projected [39]. Differently from other hyperplane-based classifiers, however, the SVM focuses its analysis on those brains (or vectors) that are more closely located to or on the hypersurface separating the two groups, which are called the “support vectors,” maximizing the distance between the nearest vectors of the two groups. Thus, a SVM classifier inherently focuses on subtle between-group morphological differences and not on gross differences that are easily identifiable [39].

For each of the two-group comparisons, the diagnostic performance of the COMPARE classifier was estimated using the leave-one-out crossvalidation (LOOCV) method. In each LOOCV experiment, one subject was first selected as a testing subject, and the remaining subjects were used for the entire adaptive regional feature extraction, feature selection, and training procedure. Then, the classification result on the testing subject using the trained SVM classifier was compared with the ground-truth class label, to evaluate the classification performance. By repeatedly leaving each subject out as a testing subject, we obtained the average classification rate from all of these LOOCV experiments [7].

After LOOCV, high-dimensional spatial maps of the brain regions that constitute the patterns of brain tissue distributions characteristics of the three SVMs were generated by COMPARE as previously described and validated [7]. This spatial feature map shows how frequently a particular region/feature was selected during all the LOOCV tests, displaying regional brain volume changes as one follows the path of the abnormality score from positive (patient-like) to negative (control-like). A scale ranging from 0 to 1 is set for each region, reflecting the relative importance for between-group discriminations based on the LOOCV experiments [7]. It is important to notice, however, that the discriminative morphological pattern generated by the classifier reflects a set of brain regions needed for between-group classification, but not necessarily all areas of regional brain volume differences between the groups under study.

### 2.4. ROC Curve Analysis

The classification scores obtained by the COMPARE analyses were evaluated using a *receiver operating characteristic* (ROC) curve aiming to visualize the diagnostic performance of the classifier in each of the pairwise comparisons and to calculate the area under the curve (AUC).

Indices of diagnostic performance such as DA (overall classification rate), sensitivity, specificity, positive predictive value (PPV), and negative predictive value (NPV) were calculated using a 2 × 2 contingency table. In the ROC curves, the individual *Z* scores obtained by the SVM classifier were plotted in a graph according to the true positive rate (*y*-axis, corresponding to the sensitivity measure) versus false positive rate (*x*-axis, corresponding to 1-specificity) generated in the group classification [40]. This procedure allowed us to adjust the threshold used by the SVM classifier according to the desired sensitivity/specificity relationship. We will report herein the sensitivity and specificity values observed when the highest classification accuracy was achieved.

The AUC measure of a classifier is equivalent to the probability that the classifier will rank a randomly chosen (truly) positive diagnosis higher than a randomly chosen negative diagnosis [40]. Thus, the AUC provides an estimate of the discriminative power of the classifier for a given condition, regardless of both the chosen threshold (classifier's score which separates the 2 groups under study) and the sample size of each group.

## 3. Results

### 3.1. Demographic and Clinical Details

Demographic and clinical data for the psychotic BD-I and MDD groups, as well as for the two subsamples of matched controls are summarized in Table 1.

More patients with psychotic MDD were using antipsychotic and antidepressant agents at the day of MRI scanning relative to the BD-I group, whereas more individuals with BD-I were taking mood stabilizers. Also, 3 MDD patients were left-handed, whereas all BD-I individuals were right-handed (Table 1).

### 3.2. Diagnostic Performance of the Classifier

**Figure 2:**
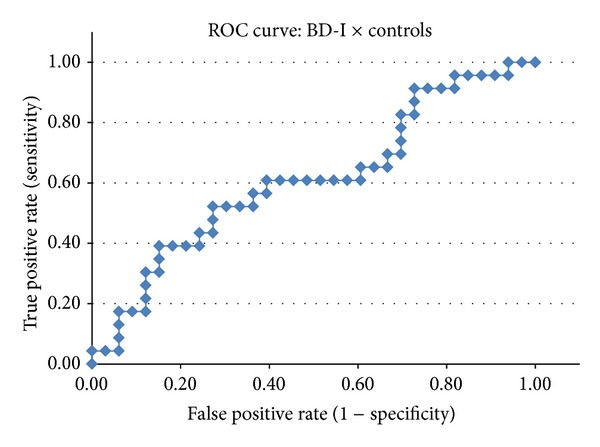
ROC curve for the comparison between bipolar I disorder (BD-I) individuals and healthy controls.

**Figure 3:**
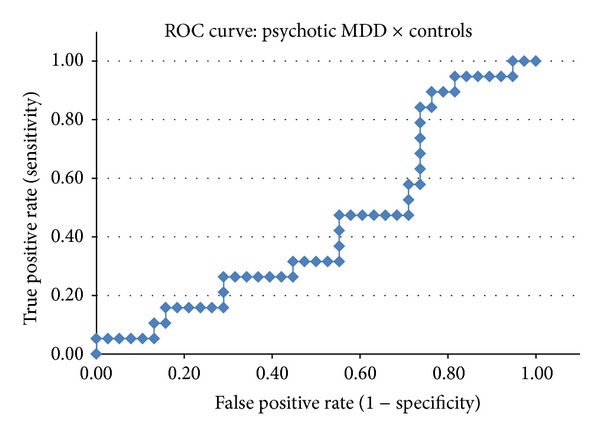
ROC curve for the comparison between patients with psychotic major depressive disorder (MDD) and healthy controls.

**Figure 4:**
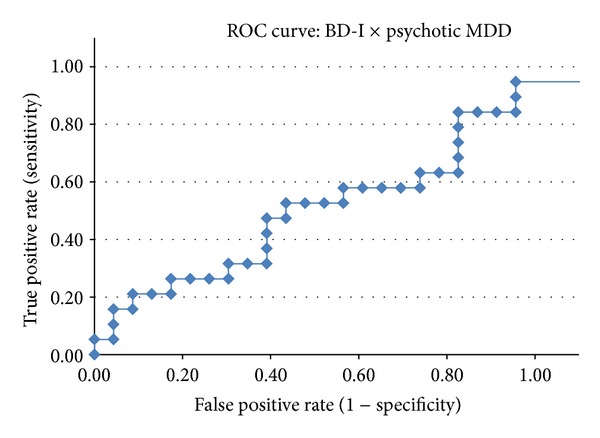
ROC curve for the direct comparison between patients with psychotic bipolar I disorder (BD-I) and psychotic major depressive disorder (MDD).

Table 2 shows the measures of diagnostic performance for the three pairwise comparisons: psychotic BD-I versus controls, psychotic MDD versus controls, and psychotic BD-I versus psychotic MDD. The ROC curves for each of these comparisons are depicted in Figures 2, 3, and 4 (resp.).

The SVM classifier attained poor discrimination in the pairwise comparisons between first-episode of psychotic mania versus controls (DA = 66.1%) (Table 2 and Figure 2), and first-episode of psychotic MDD versus controls (DA = 59.6%) (Table 2 and Figure 3). The direct comparison between the BD-I and MDD groups also resulted in a classification rate near to chance (DA = 54.76%) (Table 2 and Figure 4).

## 4. Discussion 

To our knowledge, the present study is the first to apply a SVM classifier to conventional structural (T1-weighted) MRI data of first-episode patients with BD-I and psychotic MDD using an epidemiologic approach to recruit both patients and controls.

In regard to the individual classification of patients with BD-I (first-episode of psychotic mania) and psychotic MDD, the negative results obtained suggest that neuroanatomical pattern classifiers based solely on structural MRI images possess poor diagnostic power to discriminate BD-I and psychotic MDD cases from controls, as well as from each other, at least at an early course of their illnesses. The fact that a relatively high number of morphological features were used for each pairwise classification (i.e., 53, 80, and 99) compared to previous studies using the same method but achieving better between-group discrimination reinforces this notion. That is, the classifier failed to find a specific pattern that affords good separation between the study groups and each of these features contributes very little to the classification analyses.

Congruently with our results, the few studies with structural MRI and neuroanatomical pattern classifiers in mood disorders published to date have achieved lower DA than fMRI studies [17–21]. Also, the literature on structural MRI investigations of BD has consistently shown a great variability of findings, including many negative studies and low reproducibility even across the different meta-analyses published so far [41–43]. Thus, it is conceivable that such inhomogeneity denotes that structural brain abnormalities in mood disorders remain very subtle to be detected by current neuroimaging techniques and cannot provide a reliable frame to automated classification methods. Conversely, recent studies using neuroanatomical pattern classifiers to evaluate fMRI datasets of previously medicated patients with chronic depressive disorders have shown promising results [8, 10], which suggests that functional neuroimaging measures might afford better discrimination of mood disorders cases than structural MRI. Nonetheless, incipient results of fMRI studies that attempted to discriminate BD from MDD using pattern classifiers are conflicting [14–16].

The adequate selection of relevant features for between-group discrimination is one important methodological step of neuroanatomical pattern classification studies [7, 44]. However, the stability of the model generated by the classifier and how generalizable this model is to the full range of patients suffering with a given disorder in the general population relies heavily on an adequate sample size [45] and also on the method employed for recruitment of cases and controls for the study [6, 46]. Population-based designs are likely to reduce selection biases by ensuring that control individuals represent the population from which the cases came from, therefore providing a valid estimate of the exposure of interest in that population [6, 23, 46, 47]. This is particularly important for the aim of developing a neuroimaging tool to aid in diagnostic and prognosis evaluations in clinical psychiatric practice, as “real world” patients present with a range of clinical comorbidities (such as substance use disorders) and variable disease courses [6, 46]. In this regard, it is interesting to notice that our group has previously used the COMPARE classifier in the first-episode SZ arm of the original population-based investigation from where the present samples of affective patients were drawn [6]. In that study, we found an overall modest DA of 73.4% in the individual discrimination between first-episode SZ (*n* = 62) and HC (*n* = 62), which is lower than the DA reported by most preliminary studies that have applied neuroanatomical classifiers in samples of SZ patients selected in academic institutions [6] but similar to that reported in the large, representative SZ sample recruited by Nieuwenhuis et al. [45].

There are a number of methodological limitations that should be weighted in the interpretation of our results. Firstly, a significant proportion of our BD-I and MDD patients (43.5% and 78.9%, resp.) were using antipsychotic medication at the day of MRI scanning. Although the time of such exposure was relatively short, it is known that antipsychotic treatment is associated with both GM and WM reductions [41, 48] and, thus, might have influenced our results. Secondly, comorbid substance abuse or dependence is another important confounding variable in the assessment of regional brain volumes [32] and the fact that a substantial proportion of the patients enrolled in our study presented a positive history of substance misuse could have limited the sensitivity of the classifier to identify morphometric abnormalities specifically associated with BD-I and psychotic MDD diagnoses. Nevertheless, substance misuse is pervasive in mood disorders, and a useful classifier should discriminate patients despite such comorbidity. Finally, the size of the BD-I and psychotic MDD groups may have been insufficiently large to avoid the risk of type II errors. Thus, more studies with larger samples of BD and psychotic MDD patients are needed in order to further confirm the results observed in this proof of concept investigation.

## 5. Conclusion

Neuroanatomical pattern classification is a recent method that affords individual classification of brain measures and, thus, is considered promising for developing a tool to improve diagnostic accuracy in the psychiatric practice. However, in the present structural MRI study, the diagnostic performance of such method in the discrimination between psychotic MDD, BD-I, and HC was limited. New studies preferably with larger samples are warranted to further confirm that classifiers based solely on structural MRI scans do not achieve satisfactory discrimination of individuals with mood disorders.

## Figures and Tables

**Table 1:** Demographic and clinical information for patients with first-episode of psychotic mania (BD-I), psychotic major depression (MDD), and subsamples of matched healthy controls (HC).

	BD-I	HC 1	MDD	HC 2	Statistical tests
	(*n* = 23)	(*n* = 33)	(*n* = 19)	(*n* = 38)
Age (mean ± SD)	27.09 ± 8.87	27.55 ± 6.41	29.05 ± 8.34	29.66 ± 7.92	*t* = 0.734, df = 40, *P* = 0.467
Gender (number of males; %)	9 (39.1%)	13 (39.4%)	4 (21.1%)	8 (21.1%)	*χ* ^2^ = 1.59, df = 1, *P* = 0.207
Handedness (number of right-handed; %)	23 (100%)	32 (97.0%)	16 (84.2%)	35 (92.1%)	**χ** ^2^ **= 3.91, df = 1, *P* = 0.048**
Substance misuse^a^	7 (30.4%)	—	3 (15.8%)	—	
Duration of illness (days; mean ± sd)	184.5 ± 130.7	—	250.8 ± 205.7	—	Mann-Whitney test, *P* = 0.441
Duration of untreated psychosis (days; mean ± sd)	44.3 ± 57.2	—	43.0 ± 48.3	—	Mann-Whitney test, *P* = 0.595
Medication use at the MRI (*n*; %)					
Antipsychotics	10 (43.5%)	—	15 (78.9%)	—	**χ** ^2^ **= 5.43, df = 1, *P* = 0.020**
Mood stabilizers^b^	12 (52.2%)	—	4 (21.1%)	—	**χ** ^2^ **= 4.27, df = 1, *P* = 0.039**
Antidepressants	1 (4.3%)	—	10 (52.6%)	—	**χ** ^2^ **= 12.54, df = 1, *P* < 0.001**

BD-I: bipolar I disorder (FE mania); MDD: major depressive disorder; HC 1: subsample of healthy controls selected for the comparison with BD-I patients; HC 2: subsample of healthy controls selected for the comparison with patients with psychotic MDD; MRI: magnetic resonance imaging.

^a^Number of patients with a positive diagnosis of DSM-IV substance use disorder (prevalence).

^b^Lithium, carbamazepine, and/or sodium valproate/divalproex.

We have set in bold the results that present statistical difference.

**Table 2:** Diagnostic performance of the SVM classifier in the individual discrimination of cases of BD-I and MDD with psychotic features versus controls.

Pairwise comparison	AUC^a^	Accuracy	Morphological features^b^	Sensitivity	Specificity	PPV	NPV
Psychotic BD-I (*n* = 23) ×	0.61	66.1%	99	39.1%	84.8%	64.3%	66.6%
Matched controls (*n* = 33)							

Psychotic MDD (*n* = 19) ×	0.44	59.6%	80	31.6%	73.7%	37.5%	68.3%
Matched controls (*n* = 38)							

Psychotic BD-I (*n* = 23) ×	0.52	54.76%	53	57.9%	52.1%	50.0%	60.0%
Psychotic MDD (*n* = 19)							

BD-I: bipolar I disorder (first-episode mania); MDD: major depressive disorder; PPV: positive predictive value; NPV: negative predictive value.

^a^Area under the curve; ^b^number of morphological features used for the best classification rate (accuracy).
